# Whole-Genome Sequencing and Characterization of Buffalo Genetic Resources: Recent Advances and Future Challenges

**DOI:** 10.3390/ani11030904

**Published:** 2021-03-22

**Authors:** Saif ur Rehman, Faiz-ul Hassan, Xier Luo, Zhipeng Li, Qingyou Liu

**Affiliations:** 1 State Key Laboratory for Conservation and Utilization of Subtropical Agro-Bioresources, Guangxi University, Nanning 530005, China; Saif_ali28@yahoo.com (S.u.R.); luoxier92@163.com (X.L.); zp.li@gxu.edu.cn (Z.L.); 2 Institute of Animal and Dairy Sciences, Faculty of Animal Husbandry, University of Agriculture, Faisalabad 38040, Pakistan; f.hassan@uaf.edu.pk

**Keywords:** buffalo, evolution and domestication, genome sequencing advancement, candidate genes, traits

## Abstract

**Simple Summary:**

Recent advancements in high-throughput technologies like whole-genome sequencing, genome-wide association study (GWAS), gene expression profiling, next-generation sequencing (RNA and DNA), and genome-wide CHIP-seq scanning are used to detect genetic variants and study gene regulation, gene functioning, and single nucleotide polymorphism (SNP) ordering resources. These techniques offer a wide range of whole-genome data and high coverage to genomic, epigenomic, transcriptomic, and proteomic information related to cellular interactions, functioning, and behavior. In buffaloes, candidate gene studies use the available genetic resources to uncover the functional candidate genes and their interactions associated with buffalo productivity, including production, adaptation, and disease resistance. Thus, the whole-genome and candidate gene approach to next-generation data could help elucidate the inheritance of complex traits, full genomic coverage, and the genetic dissection of productivity-related attributes, which could ultimately help accelerate genetic progress in buffaloes.

**Abstract:**

The buffalo was domesticated around 3000–6000 years ago and has substantial economic significance as a meat, dairy, and draught animal. The buffalo has remained underutilized in terms of the development of a well-annotated and assembled reference genome de novo. It is mandatory to explore the genetic architecture of a species to understand the biology that helps to manage its genetic variability, which is ultimately used for selective breeding and genomic selection. Morphological and molecular data have revealed that the swamp buffalo population has strong geographical genomic diversity with low gene flow but strong phenotypic consistency, while the river buffalo population has higher phenotypic diversity with a weak phylogeographic structure. The availability of recent high-quality reference genome and genotyping marker panels has invigorated many genome-based studies on evolutionary history, genetic diversity, functional elements, and performance traits. The increasing molecular knowledge syndicate with selective breeding should pave the way for genetic improvement in the climatic resilience, disease resistance, and production performance of water buffalo populations globally.

## 1. Introduction

The buffalo was domesticated around 3000–6000 years ago and has substantial economic significance as a meat, dairy, and draught animal [1,2,3]. This species is stereotypically distributed across wet grasslands, tropical and subtropical forests, swamps, and marshes. Though buffaloes are terrestrial mammals, they spend much of their time wallowing in rivers or mud. Wallowing is a comfort behavior that not only helps the animals to keep cool but also protects them from insect bites. Typically, buffaloes are inhabitants of mire holes, rivers, streams, trees, and tall grasses that provide enough food, water, and coverage [2]. *Bubalus* is thought to have spread from Europe to southern Asia in the Pleistocene epoch, but a progressive dry climate became the ultimate reason for contracting its distribution area to Indonesia, India, and Southeast Asia. It is believed that the buffaloes were brought to Italy from central Europe in the 6th century or from the Gulf of Tunis in the 7th century, along with Arab conquests. There is, however, recent evidence of the introduction of buffaloes into Africa, America, and Australia [1,3].

**Figure 1:**
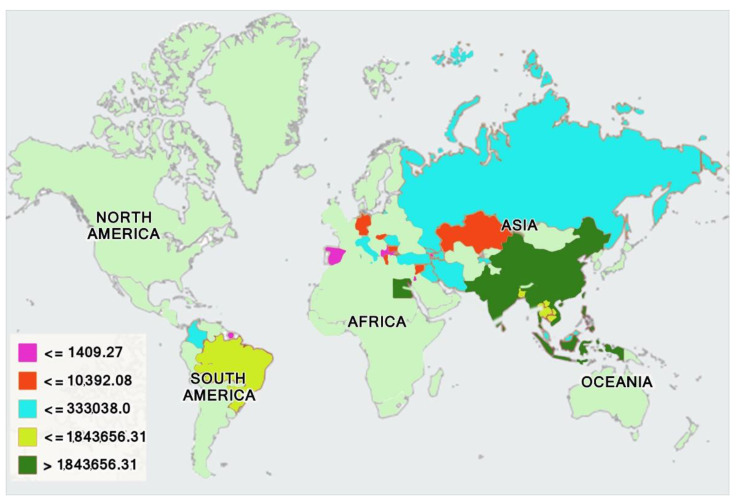
Geographical distribution of buffalo population [4].

The worldwide buffalo population is approximately 207 million heads, with a distribution of more than 200 million (97%) in Asia, 6.831 million (2%) in Africa (predominantly Egypt), 1.449 million (0.7%) in South America, and less than 0.144 million (0.2%) in Australia and Europe, as shown in Figure 1 [4]. Buffaloes constitute a littl more than 11.1% of the global bovid population, but larger human communities depend on buffaloes for their livelihood as compared to any other domesticated species [1]. Globally, in the last 20 years, about a 2% annual increase in the buffalo population has been observed. Buffaloes contribute about 13% of the global milk supply [5]. Buffalo milk contains a lower amount water but higher amounts of protein, fat, minerals, and lactose as compared to cow’s milk [2,4].

Buffalo milk is considered more suitable for the production of butter, cheese, and other high-quality dairy products. Buffalo meat is lean with lower cholesterol and fat contents than beef and comparatively better taste [2]. In waterlogged conditions, like rice paddy fields, buffaloes are considered superior draught animals and have been frequently used for ploughing and can drag heavier loads than other cattle. Furthermore, their hide is quite useful in making a variety of leather products [2,4]. Buffalo manure is used as a natural fertilizer, which supplements the soil with organic matter and essential elements and successfully reduces the need for chemical fertilizers. Most importantly, in villages, the small farmers and poor people prefer to rear buffaloes owing to their strong ability to efficiently use low-quality and less digestible roughage than other ruminants, which makes them easy to raise on locally available crop residues. Additionally, buffaloes are considered lucrative assets and ready cash, particularly for landless and smallholder families [6].

Buffaloes are a comparatively resilient and thrifty animal, but it is also vulnerable to parasites and diseases that affect cattle, including tuberculosis, piroplasmosis, brucellosis, trypanosomiasis, and rinderpest [6]. Owing to wallowing behavior, buffaloes are less susceptible to ectoparasites, like ticks, that cause diseases such as babesiosis, anaplasmosis, and theileriasis [4] and also resist the screwworm fly, which is the main pest of farm animals. After a buffalo has wallowed, the mud covering the buffalo’s body coat suffocates the screwworm fly larvae, ultimately providing protection from screwworm fly larval infection [6]. Though wallowing behavior helps to resist some parasites, it increases susceptibility to liver fluke, as buffaloes are more vulnerable to the waterborne stage of liver fluke. Furthermore, buffaloes are less affected by mastitis than dairy cattle due to wallowing behavior [6].

Buffaloes are adaptable and productive domestic animals, having national and international importance and contributing significantly to the rural economy of many countries [7]. As a species, buffaloes have remained underutilized despite having many comparative advantages, as mentioned above. The major reasons behind this underutilization include many problems and challenges, such as a higher incidence of infertility, deprived reproductive efficiency, poor production potential, and lesser calf survival rates [8]. In spite of their major contribution to the livestock sector, the development of buffaloes as a dairy producer is handicapped, mainly due to delayed puberty [9], poor estrus expression [10], a distinct seasonal reproductive pattern [11,12], and prolonged calving intervals [13]. Moreover, quite a few calving disorders negatively affect the reproductive and productive performance of buffaloes [14].

This scenario requires intensive research work on buffalo genetic resources to improve and further develop available buffalo germplasm to enhance buffalo production and effective use. Genome-based research has created a wide array for endorsing and employing gene technologies in different areas of livestock production. Genome biotechnology provides an opportunity to develop sustainable animal production systems by manipulating the intra- and interbreed genetic diversity. Genomic characterization is a preliminary step required to differentiate molecular phenotypes and predict breeding values to screen superior mates to produce improved progeny. Buffaloes are a globally important genetic resource that need to be characterized at a genomic level, which necessitates the development of a well-annotated and assembled reference genome de novo for buffaloes because it is mandatory to explore genetics and understand biology to devise breeding strategies and perform the genomic selection.

The present review focuses on providing comprehensive insight into the available genomic resources and genome re-sequencing efforts related to buffaloes aimed to better understand buffalo physiology, which would help to optimize reproduction efficiency, production potentials, nutritional value, and product quality.

## 2. Buffalo Genome Architecture

The buffalo is an even-toed hoofed animal of the Bovidae family, genus *Bubalus*, and tribe Bovini. The wild buffalo has two major types: the Asian buffalo (*Bubalus bubalus*) and the African buffalo (*Syncerus caffer*) [15]. The African buffalo is of two types: *Syncerus caffer nanus* (2*n* = 54) and *Syncerus caffer caffer* (2*n* = 52) [16], whereas *Bubalus bubalis carabanesis*, with karyotype 2*n* = 48, and *Bubalus bubalis bubalis* (2*n* = 50) are two types of Asiatic buffalo (*Bubalus bubalis*). Generally, it is assumed that the domesticated water buffalo (*Bubalus bubalis*) originated from the wild buffalo (*Bos arnee*) that was found in the northeastern region of India [15].

### 2.1. Chromosomal Array of Asian Buffaloes

**Figure 2:**
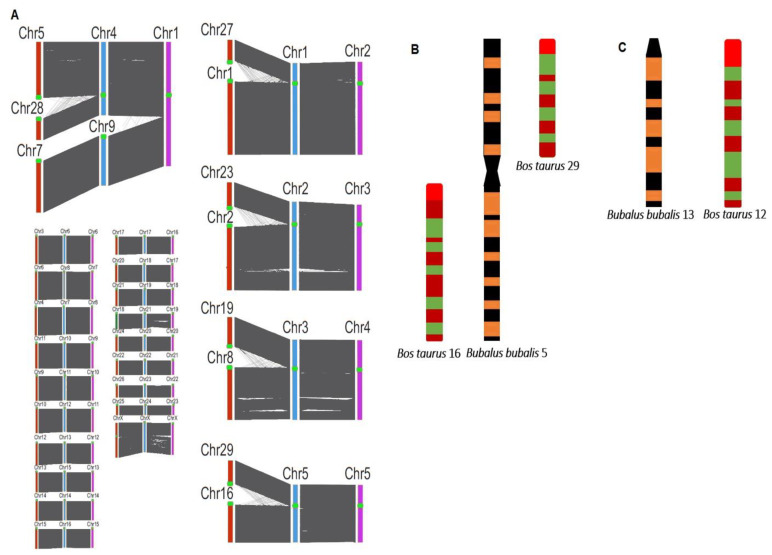
Cytogenetics of buffaloes (river and swamp) and cattle (*Bos taurus*). (**A**) Synteny analysis of buffaloes (river and swamp) and cattle. The cattle chromosomes are presented in red, river buffalo chromosomes in blue, and swamp buffalo chromosomes in pink and centromeres in red dots [21]. (**B**,**C**) The similar banding pattern of different chromosomes of buffaloes and cattle [20].

The chromosomal array presents 25 pairs of chromosomes in river buffaloes (*Bubalus bubalis bubalis*), whereas swamp buffaloes (*Bubalus bubalis carabanesis*) have 24 chromosome pairs. Genetic diversity in the two buffalo subspecies, the swamp buffalo and the water or river buffalo, is spotted by the fusion of chromosomes 4 and 9 to chromosome 1 in swamp buffalos, while all the other chromosomes and chromosomal arms are conserved in both subspecies (Figure 2) [17]. A recent study demonstrated that buffalo submetacentric chromosomes are a centric fusion of ancestral acrocentric chromosomes [18]. Furthermore, the hybrid of the two subspecies is fertile but has 49 chromosomes and in subsequent mating has lower reproductive capability. All the chromosomal pairs, including the sex chromosomes, are acrocentric in river and swamp buffaloes except for the first five chromosomal pairs, which are bi-armed (Figure 2). Various studies have reported that domestic cattle and buffaloes, members of the Bovidae family, are closely linked. The corresponding chromosome in buffaloes and cattle can be matched from arm to arm (Figure 2) as cytogenetic characterization presents chromosomal banding and gene ordering homology in both members (Figure 2) [17,19].

For instance, there are 29 acrocentric chromosomal pairs together with a pair of sex chromosomes (XY) in the cattle genome, whereas the buffalo genome consists of 19 acrocentric and 5 bi-armed chromosomes, in addition to a pair of sex chromosomes (XY) (Figure 2). In cattle, two acrocentric chromosomes fuse to form the corresponding first five bi-armed chromosomal pairs of buffaloes (i.e., chromosomes 1 and 27 fuse to form buffalo chromosome 1, 2 and 23 fuse to form 2, 8 and 19 fuse to form 3, 5 and 28 fuse to form 4, and 16 and 29 fuse to form 5). All other buffalo chromosomes correspond to each of the remaining cattle chromosomes [18,19,20,21,22]. Synteny sequence confirmed the five chromosomal fusion events in cattle and river buffaloes (resulting in five bi-armed autosomal pairs of chromosomes), as shown in Figure 2A. Precisely, the chromosomal fusion of river buffalo chromosomes 4 and 9 results in the longest chromosome in swamp buffaloes (Figure 2A) [21].

### 2.2. Chromosomal Array of African Buffaloes

Cytogenetic studies have demonstrated that *S.c. caffer* has 26 pairs of chromosomes and *S.c. nanus* possesses 27 chromosome pairs. Both species can interbreed, while their offspring have 53 chromosomes, with a lower fertility rate due to gamete imbalance, which results in abnormal zygote development [17]. The major difference between the two African buffalo subspecies is the presence of three bi-armed chromosomes in *S.c. nanus* whereas *S.c. caffer* has four chromosomes. All the rest of the chromosomes, plus the sex chromosomes, in both subspecies are acrocentric. These bi-armed chromosomal pairs in *S.c. caffer* were created through the fusion of cattle chromosomes 1 and 13, 2 and 3, 5 and 20, and 11 and 29 [23].

Additionally, no bi-armed chromosome pair sharing between *Bubalus* and *Syncerus* is detected, which suggests that there are no crosses between these two genera and if it happens, the resulting hybrid would have a deranged set of chromosomes. Consequently, the chromosome morphology categorizes each as a separate genus [17].

### 2.3. Sex Chromosomes

A high degree of homology has been revealed in different studies among bovid autosomal chromosomes, chromosomal arms, banding patterns, and the order of genes in buffaloes, cattle, goats, and sheep [24,25]. The bovid sex chromosomes have a more complex sequence rearrangement as compared to the very similar autosomal chromosomes [17]. Comparing the chromosomal bands that display conserved portions of these chromosomes, large blocks of constitutive heterochromatin are in X-chromosomes of buffaloes while cattle lack this. Cytogenetic studies have illustrated complex rearrangements of loci order on sex chromosomes that appeared in the karyotype evolution of buffaloes and cattle. X-chromosomes of both buffaloes and cattle have a variable position of the centromere with the same gene order (Figure 2). This indicates the constitutive heterochromatin loss in cattle that distinguished it from buffaloes and, eventually, the centromere translocation event [17]. Comparative FISH mapping demonstrated the analogous positions of the Y-chromosomes in buffaloes and cattle. The differences observed in the Y-chromosomes of buffaloes and cattle are pericentric inversion (an inversion with the centromere and both arm breakage points) and that the Y-chromosome of buffaloes has more heterochromatin and is larger than the Y-chromosome of cattle [17].

## 3. Evolution and Domestication of Buffaloes

Previous studies have reported that the divergence time of swamp and river buffaloes ranges between 10,000 years and 1.7 million years [1,26,27,28,29,30,31,32,33]. This variation is possibly due to the differences in the population size, source of samples, mitochondrial DNA (mtDNA) sequences, and rate of mutation. Whole mitogenomes have been employed to estimate the time of divergence, which ranged from 860,000 to 900,000 years [34], which is perhaps overestimated as population splitting time may have been much earlier based on mtDNA divergence [35]. Recently, a whole-genome study evaluated the divergence time between the river and swamp buffaloes by using Ks distribution and MCMC tree approaches and reported the estimated divergent time as 1.1 MYA and 3.5 MYA, respectively [21].

However, it is interesting to hypothesize that the estimated divergence time is the period when the Arakan Mountain system (east and west) separated the panmictic population into two regions; subsequently, chromosomal translocation occurred and diffused through the population of eastern *B. arnee*. Lau et al. [30] speculated that the *B. arnee* species originated in Southeast Asia mainland and afterward spread to the west Indian subcontinent, where river-type animals evolved. Instantly, the 4p/9 chromosome admixture occurred in the Southeast Asian mainland population. The Pleistocene glacial events affected the ancestral population of swamp buffaloes, which led to a decline in population size and development of remote refugia [34] and probably the river buffalo ancestors were also affected similarly.

During Holocene, the postglacial period (6000–11,000 years), the population size expanded and genetic data have indicated the independent event of domestication of swamp buffaloes in Southeast Asia and the river types in the Indian subcontinent [3,21,30,31,32,36,37]. Molecular data provide the evidence that the divergence of swamp and river buffaloes preceded their domestication [36,38], where the swamp buffalo was domesticated in the Indo–China bordering area about 3000–7000 years ago and the river buffalo around 6300 years ago in the west of the Indian subcontinent [34,39,40]. Rapid population expansion and geographical distribution during the postglacial phase (Holocene) after domestication occurred due to climate improvement [34,41]. Historical and archaeological data have proved the westward migration of the river buffalo from its domestication epicenter to areas as far away as the Balkans, Italy, and Egypt [42].

**Figure 3:**
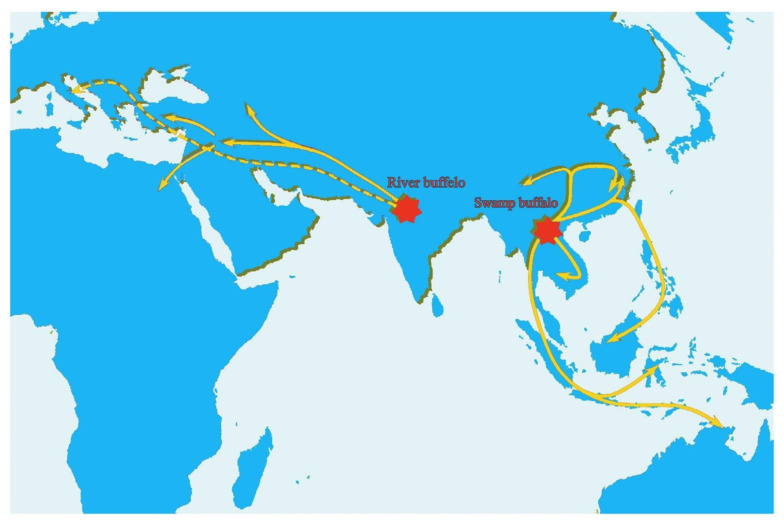
A proposed pattern of riverian and swamp buffalo migration. The dashed arrow points to an initial and independent migration way that might have led river buffaloes into Europe [45] (figure reproduced by Rehman et al., 2021, with the permission of authors).

Studies have confirmed this geographical expansion of buffaloes using molecular data, including SNPs and mtDNA [38], and archaeological evidence [37]. Generally, it looks like successive events of migration occurred at a geographical scale at different times. However, the buffalo population prevalent in the geologically adjoining areas of Pakistan and Iran are genetically different, as the population of Iran is closer to that of Turkey and Egypt. The Egyptian and Italian buffalo populations are also different from each other. Thus, Zeuner’s [43] suggestion looks promising that the westward migration remained discontinuous, late, and slow. Colli et al. [37] proposed two independent events of migration that are well matched with population genetic diversity: the first one the proto-Mediterranean gene pool of Italy that came through the Balkans and the second the proto-Middle Eastern gene pool toward the Caspian Sea and Mesopotamia, later extended to Egypt and Turkey (Figure 3) with the expansion of Islam, as proposed by Unal et al. [44].

Microsatellite, mtDNA and skeletal fossils data of the domesticated buffalo suggest that Indo-China, south China, and north Thailand are the regions where swamp buffaloes were domesticated and spread to the Indonesian islands (Sulawesi, Sumatra, and Java) through Malaysia and then through northeast/north to central China and Philippines and Borneo through the eastern island route via Taiwan [39,40,46].

**Figure 4:**
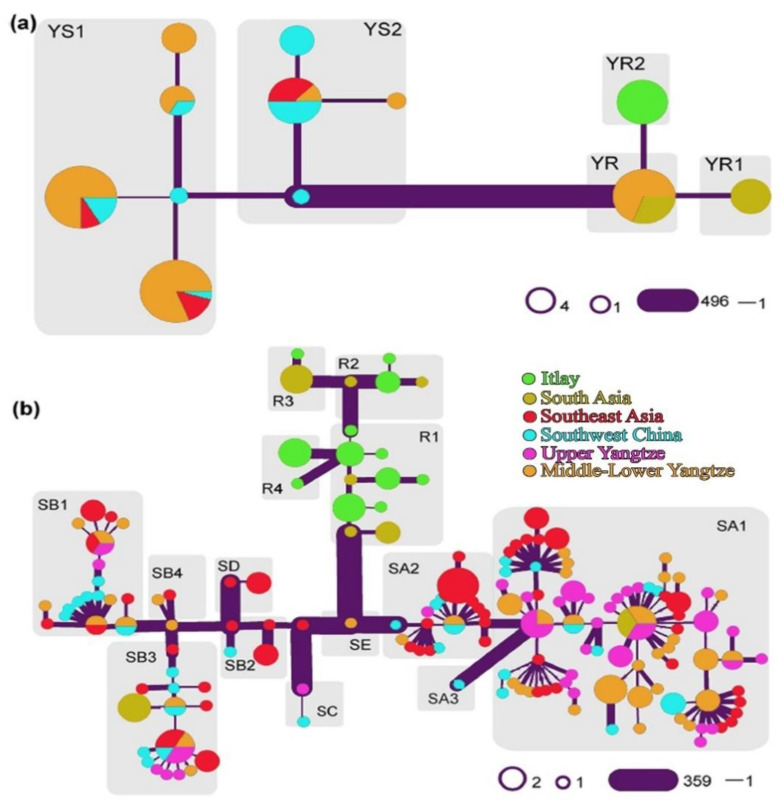
Phylogenies based on mitogenome and Y-chromosome. The pairwise differences between the adjoining haplotypes are represented by the widths of the edges. (**a**) The network of Y-chromosome using 520 SNPs. (**b**) Swamp buffalo mitogenome network. Diverse haplogroups of mitogenomes and Y-chromosomes are characterized in each gray box [47] (figure reproduced by Rehman et al., 2021, with the permission of authors).

Sun et al. [47] described Y-chromosome-based distinctive riverian buffalo lineages with a base group (YR) unique to Pakistan and Indian breeds. The YR1 haplotype was a typical inhabitant of south Asia, but YR2 was only prevalent in Italy. The previous study-based scenarios indicated the likelihood early domestication of river buffaloes in the Indo-Pakistan region, followed by their subsequent migration to Europe [37]. Afterward, the YR1 group remained in south Asia, though it still is extremely diffused, while the YR2 buffalo group became unique to Italy. In the case of swamp buffaloes, the haplogroup YS2 is distributed across Southeast Asia and southwest China (84.62%), whereas the YS1 buffaloes are dominating in the lower-middle and upper regions of Yangtze (76.09%). The early divergence of YS2 in the phylogeny indicates that the swamp buffalo population traveled from the south to the northern regions and YS1 experienced population expansion [47]. From the swamp buffalo mitochondrial gene pool, a pattern of geographical events has also been identified. Taking together the frequencies of swamp buffalo uniparental haplogroups as represented in Figure 4, a correlation between mtDNA and Y-chromosome haplogroups, i.e., between SA and YS1 and between SB and YS2 is observed, which points out the similarities between paternal and maternal histories.

Mitochondrial genome-based reports anticipated two sub-populations of swamp buffaloes [39], while the whole-nuclear-genome study suggested a monomorphic population of swamp buffaloes [21]. Other domesticated animals, including pigs, horses, and dogs, also followed the same phenomenon, representing hybridization in the past [48]. Furthermore, the time of divergence between the river and swamp buffaloes determined by Luo et al. [21] overlapped well-known geographical events, providing a hypothetical explanation for the forces responsible for this divergence. Particularly, in the Xixiabangma glacial era, the descending sea-level created a migration passage for ancient buffaloes to cross the India–Myanmar bordering area, so initiating geographical separation and facilitating the fixation of genetic polymorphisms and chromosomal number variation into the genomes of two isolated populations. So, buffalo migration patterns, i.e., in Yunnan, Laos, and Myanmar, developed a hybrid zone harboring a genetic admixture of river and swamp buffaloes. This proposed scenario indicates a higher flow of genes among the buffalo population of the Southeast Asian region [21].

## 4. Recent Advances in Whole-Genome Sequencing of the Buffalo Genome

Buffaloes remained underutilized despite their excellent genetic potential. To make the best use of the buffalo, it is imperative to characterize genetic diversity and study its genomic architecture through high-throughput technologies. During the last five years, marvelous advancement in whole-genome sequencing of buffaloes has been made. As of March 14, 2021, a total of five annotated buffalo genome assemblies had been deposited in the National Center for Biotechnology Information (NCBI) [49]. A total of 1,921,573 nucleotide and 34,831 water buffalo gene sequences, including 471 mitochondrial sequences, have been submitted in the GenBank database. The nucleotide sequences deposited in GenBank include 273,061 whole-genome shotgun sequences, 538 microsatellite, 490 minisatellite, and 503 satellite sequences, while other sequences mainly consist of cytosine-phosphate-guanine (CpG) island, gene-like sequence cis-acting regulator, and repeat sequence and there are some additional genomic sequences.

Mintoo et al. [50] re-sequenced and assembled the river buffalo genome (2*n* = 50) from Bangladesh. The size of the genome was 2.77 Gb, with a scaffold N50 of 6.9 Mbp and contig N50 of 25 kb. From the assembled genome, 24,613 genes were annotated for further functional genomic studies. The assembled genomes were comparable with those of the water buffalo (Mediterranean) and the African buffalo. They used two different strategies to evaluate the genome completeness. Firstly, they downloaded EST/mRNA sequences from the NCBI and aligned them with the assembled genome by BLAST [51], which provided well-aligned data of approximately 98.15%. Secondly, they employed benchmarking against universal single-copy orthologs (BUSCO 2.0), which gave 94.3% core genes coverage from the assembled genome. More CpG island sequences were identified in the water buffalo genome (39,578) as compared to the cattle genome (12,120) [52]. This variation is attributed to the differential rate of recombination and chromosome size of these two species [53]. Mintoo et al. [50] stated segmental duplication of the buffalo genome, 94.5 Mbp which is comparable with about 94.4 Mbp of a previously reported cattle sequence [54], suggesting an event of duplication in the last common ancestor of cattle and water buffaloes [55]. In water buffaloes, 51.19% of the genome accounts for repetitive DNA (1418 Mbp) sequences, which is ~13% higher than that of African buffaloes (37.21%) [56]. Notably, 49.06% of the genome were transposable elements (TEs), of which 41.50% accounted for long interspersed nuclear elements (LINEs) as the core TE component. Additionally, 38,483 transfer RNAs (tRNAs), 867 ribosomal RNAs (rRNAs), 1758 smalls nuclear RNAs (snRNAs), and 23,310 microRNAs (miRNAs) were annotated in the water buffalo genome. Moreover, expansion of 159 gene families was observed substantially in water buffaloes in contrast to other mammals [50]. A study also identified 382 candidate genes with positive selection sites that might play a role in the development of climatic adaptability in water buffaloes in different environments [50]. Mostly these genes were annotated to the metabolic pathways, the immune system functional pathway, and signal transduction pathways [50].

Recently, Dutta et al. [57] re-sequenced the genomes of 73 animals from six distinct buffalo breeds (Bhadawari, Banni, Murrah, Jaffarabadi, Surti, and Pandharpuri) to evaluate the genetic diversity of the water buffalo population in India by comparing them with 6 Mediterranean buffaloes as a distinct outgroup. Half of the animals from each of the breeds were sequenced at an average of 8× and the remainder at 37×, while all the Mediterranean buffaloes were sequenced at the coverage of a mean depth of 36×. The subsequent sequences of DNA were aligned with chromosome-level high-quality reference assembly of the water buffaloes (UOA_WB_1). Here, they identified 5,897,230 short insertions/deletions and 37,682,631 single nucleotide variants (SNVs). After the removal of low-quality variants, a final set of biallelic SNVs (26,247,559) was perceived of which 25,513,085 were autosomal [57].

The most comprehensive study on the buffalo genome to date re-sequenced 230 individuals (98 river buffaloes and 132 swamp buffaloes) across Europe and Asia [21]. Multiple approaches were used for sequencing and assembling the river and swamp buffalo reference genomes. The genomes of one Fuzhong swamp buffalo (female) and one river buffalo (Murrah female) were sequenced, and PacBio assemblies were constructed into contig N50 sizes of 3.1 Mb and 8.8 Mb for river and swamp buffaloes, respectively. The Illumina high-throughput/high-resolution chromosome conformation capture sequencing (Hi-C) was used to develop the scaffolds from contigs. These were developed into chromosomal-level scaffolds with N50 sizes of 116.1/117.3 Mb, the largest one 198.8/269.09 Mb for river/swamp buffaloes. The final assemblies covered 25 chromosomes, with 96.5% genome coverage, of the river buffalo, having an error rate of 2.13 × 10^−5^, and 24 chromosomes of the swamp buffalo, with 97.5% genome coverage, and an error rate of 9.22 × 10^−6^. The coverage and mapping rates for both buffaloes from Illumina reads reached levels >99%. Further, the repetitive sequences in the genomes of river and swamp buffaloes were 46.37% and 46.62%, respectively (Table 1), with a similar percentage of TE and ruminant-specific repeats as observed for cattle. No obvious chromosome fusion, breakpoint, or gene fusion events other than 4p/9 chromosome fusion were detected. Luo et al. [21] reported 20,202 and 19,279 gene models corresponding to river and swamp buffaloes by using repeat-masked genomes. BUSCO evaluations specified high-quality genomes with gene structure predictions representing 96%/96.8% completeness (Table 1) and identified 17,890 gene families. They also reported 78 and 99 positive selection genes in river and swamp buffaloes, respectively. In comparison to the *Bos* genus, *Bubalus* displayed more gene family contraction events (2117 vs. 1022) and fewer gene family expansion events (112 vs. 148). In the case of the two buffalo subspecies, 261 vs. 179 gene expansion events were identified in river and swamp buffaloes, respectively, which may have a functional effect related to the phenotypes of both buffalo subspecies [21].

## 5. Identification of Genes Affecting Important Buffalo Traits

Unraveling the possible role of genetic variants in and their physiological effects on phenotypic traits is the major challenge for genetics. To date, different studies have explored the association of genotypic variants in buffaloes, which are considerably related to heat stress, reproduction, behavior, coat color, and production traits (i.e., milk).

### 5.1. Candidate Gene Studies

#### 5.1.1. Heat Shock Protein Genes (HSPs)

Globally, heat stresses badly affect livestock production. HSPs are conserved molecular chaperones critical for protein maturation, refolding, and degradation, and in farm animals, including cattle and buffaloes, HSPs not only develop thermotolerance but also act as potential biological markers considered to measure the extent of heat stress in livestock animals [58]. Under stress conditions, HSPs are crucial for survival, protein homeostasis, and cellular responses. Molecular mass-based classification characterizes HSPs into *HSPB1*, *HSPD*, *HSPH1*, *HSP40*, *HSP10*, *HSP70*, and *HSP90* gene families. A total of 64 HSP genes were reported in buffaloes, of which 39 genes belong to the *HSP40* family; 4 to *HSP90*; 8 to *HSPB*; 10 to *HSP70*; and 1 each to *HSPH1*, *HSP10*, and *HSPD* [59]. In thermal stress conditions, the expression of HSPs distinctively increases to enhance the thermotolerance ability and serve as the first line of defense against heat shock to protect cells and tissues [60,61].

HSPs are known to have a crucial role in the regulation of immune response in buffaloes, as HSP-derived lymphocyte peptides are required to initiate/trigger the innate and adaptive immune responses [62]. The functional association of HSPs with reproductive efficiency has also been identified in different bovine species as variation in cattle *HSP40* genes has been associated with early in vitro embryonic development. Likewise, polymorphism in the *HSP70* family of cattle has also shown a potential association with observed differences in reproductive performance [63]. In different biological processes, HSPs (particularly *HSP40* and *HSP70*) have exhibited their distinctive roles in several pathological disorders, such as neurodegeneration, muscular dystrophy, viral infectious diseases, and cancer [64,65,66]. *HSP90*, through its conformation and stability, is involved in stimulating oncogenic proteins. Thus, such oncogenic signaling pathways can be suppressed via the inhibition of *HSP90* [65,66]. After heat shocks, the subsequent induction of *HSP70* is effective in the regulation of different physiological parameters, i.e., pulse, respiratory rates, and rectal temperature, of the animal [67].

Moreover, the SNPs in the genic, untranslated, and promoter regions of HSPs have shown significant association with higher milk production, heat tolerance, stress resilience, and disease vulnerability in livestock [21,68,69,70]. Furthermore, a recent whole-genome sequence-based study reported evidence of positive selection related to thermotolerance in buffaloes [21,59]. Luo et al. [21] reported HSP gene family expansion in both buffalo subspecies (river and swamp), which are stress-inducible molecular chaperones and responsible for environmental adaptation in buffaloes.

#### 5.1.2. Reproductive Physiology-Related Genes

Buffalo productivity is primarily attributed to reproductive efficiency [71]. Different studies have associated fertility with potential genetic variants in both females and males [72]. Recently, in swamp buffaloes, the gonadotropin-releasing hormone receptor (GnRHR) and luteinizing hormone beta polypeptide (LHB) have been identified to be associated with semen quality parameters, including higher sperm count and ejaculate volume [73,74]. In the cytochrome P450 aromatase (*CYP19A1*) gene, four SNPs were identified, three in exon 10 and one in 5′ UTR, which were associated with the fertility of Egyptian female river buffaloes [75]. Similarly, the Murrah buffaloes treated with melatonin exhibited variable rates of pregnancy due to melatonin receptor 1A (*MTNR1A*) gene diversity. Particularly, in animals having genotype TT in exon II (812 bp fragment) at position 72, the gene showed a high conception rate and estrus activity soon after the beginning of melatonin treatment [76]. A recent genome-wide association study (GWAS) detected and annotated 436 SNPs along with 34 indels in fertility-related 38 candidate genes of Murrah buffaloes [77].

Moreover, the candidate genes involved in the reproductive physiology of buffaloes at different stages are as follows: For age at first calving (AFC), interferon gene, including interferon-Tau (*IFN-TAU*), production in the early embryonic phase is the sign associated with maternal pregnancy confirmation in buffaloes [78]. The SNPs in the X-chromosome-mapped *SELP* gene are significantly linked with AFC as their expression levels could have considerable effects on conception outcomes [79,80,81]. For calving interval (CI), the *TPCNI* gene is involved in the spermatozoa acrosomal reaction that is necessary for fertilization and is interesting to study in view that male fertility might be related to herd performance as compared to female fertility-related genes. The fertilization ability of bulls in a population could be studied based on increased conception rates and decreased CI [82]. For number of services per conception (NSC), the increased expression of the *ABCC4* gene in the endometrium of pregnant cattle [83] and pigs [84] seems to act on prostaglandin efflux from cells and had a significant role in supporting pregnancy [83].

Buffaloes are a seasonal estrus species, and hormonal regulation has an impact on the animals’ reproductive performance [85]. Li et al. [55] used Buffalo SNP Array (90 K Affymetrix Axiom) and identified five genes related to hormonal regulation: for thyrotropin *TRHDE* [55]; for prostrate hormones *KCNMA1* [86], *TBCB* [87], and *CDH10* [88]; and for thyroid hormones *THRB* [89]. In seasonal cycles of reproduction and body weight, thyroid hormones play a vital role [90] and thyroid dysfunction is linked with infertility, anovulation, and abortion [91]. Furthermore, thyroid hormone resistance (THR) is significantly associated with *THRB* mutation [89]. The *KCNMA1* gene can enhance sexual behavior and erectile strength [92]. In buffalo granulosa cells, low expression of these hormone-related genes has been reported earlier but a lower hormone concentration may have a substantial effect on follicle growth regulation [93]. So, it could be assumed that buffalo fertility is somewhat controlled by genes involved in hormonal regulation [94].

Li et al. [94] reported five important genes, *CSGALNACT1*, *GMDS*, *NDUFS2*, *HYAL4*, and *KYNU*, related to metabolic pathways. The *CSGALNACT1* gene reportedly affects the follicular growth of buffaloes by regulating the glucose metabolism level. *CADM2* (cell adhesion molecule 2), which was detected in early follicular (granulosa cells) GCs, was associated with the reproductive performance of buffaloes [95]. The SNPs detected in *CADM2* upstream (about 143 kb) and downstream regions are linked with AFC and SCA traits. The *Nolz-1* or *ZNF503*, *MTPN*, and *KRR1* genes were perceived to be highly expressed in the GCs of buffaloes, suggesting that these may have aided in dominant follicle selection. The *Nolz-1* or *ZNF503* plays an essential role in cell invasion and promotes the proliferation of mammary epithelial cells [96] and embryogenesis [97]. Moreover, *KRR1* was stated to be related to polycystic ovary syndrome [98] and *MTPN* enhances skeletal muscle and cell growth [99] and plays an important role in the immune response by antigen recognition [100]. *IGFBP7* is highly expressed in buffalo GCs of antral follicles, and *IGFBP7* shares sequence identity with follistatin [101], which inhibits the secretion of follicle stimulating hormone (FSH) [102,103] and is critically involved in folliculogenesis and ovarian function [104,105]. Moreover, *IGFBP7* knockdown in buffalo GCs reportedly affects cell cycles, cell proliferation, production of progesterone and estrogen, and the number of apoptotic cells. Therefore, it was speculated that *IGFBP7* may be involved in follicular development and ovulation regulation [94].

In mammals, the reproduction system is regulated by the hypothalamic, pituitary, and gonad axis. Kisspeptin is an effective GnRH endogenous secretagogue that governs GnRH secretion, which ultimately drives spermatogenesis, steroidogenesis, and folliculogenesis and also activates ovulation in females [106].

#### 5.1.3. Milk Production-Related Genes

Identification of genomic regions and respective candidate genes associated with milk production traits is imperative to devise strategies for the genetic improvement of buffaloes. So far, in different buffalo breeds, 19 candidate genes associated with milk production containing a total of 47 mutations have been reported [107]. Of these 47 mutations, 4 were present in the promoter region, 24 in intronic regions, and 19 in coding regions; of these 19 mutations in coding regions, 13 were non-synonymous and caused amino acid substitution. These 19 candidate genes for milk production traits are classified into four major groups: milk yield-associated genes comprise *STAT1*, *STAT5A*, *LEP*, *MC4R*, *OXT*, *INSIG2*, *LALBA*, *BTN1A1*, *PRL*, *SCD*, and *SREBF1*; milk fat yield-related genes include *GHRL* and *A2M*; milk fat percentage-related genes include *STAT1*, *TG*, *A2M*, *DGAT1*, *GHRL*, *LEP*, *MC4R*, *PRL*, *SCD*, and *SREBF1*; and milk protein percentage-related genes include *CSN1S1*, *DGAT1*, *GHRL*, *ADRA1A*, *A2M MTNR1A*, *PRL* and *SPP1*, *INSIG2*, and *MC4R*, as shown in Table 2 [107].

Previously, the bovine GWAS SNP chip has been used for studying buffalo milk production traits [95,108,109] due to the chromosomal homology between cattle and buffaloes [110,111]. For the first time, Wu et al. [95] identified seven SNPs associated with milk yield in buffalo populations by using a bovine SNP chip (Illumina BovineSNP50 BeadChip). Later on, Venturini et al. [108] applied a high-density bovine SNP chip (777 k SNPs, Illumina Infinium BovineHD BeadChip) to study the Brazilian buffalo population. A huge number of SNPs related to the milk production traits were identified, but after multiple testing and modification, only two SNPs (present on chromosomes 15 and 20) were confirmed to have significant association with milk yield. Afterward, Affymetrix (Axiom Buffalo Genotyping Array 90 K) also released a commercial buffalo SNP chip [107].

For buffalo milk production traits, four GWASs have been conducted until now [111,112,113,114]. Briefly, two were performed on 1018 individuals of Italian Mediterranean buffalo [111,114] and two were performed on Egyptian and Brazilian buffaloes [111,112]. At least four candidate genes, estrogen-related receptor gamma (*ESRRG*), fragile histidine triad (*FHIT*), catenin delta 2 (*CTNND2*), and apolipoprotein B (*APOB*), were identified in two GWASs. In different buffalo breeds, *FHIT* and *CTNND2* were detected by a bovine SNP chip [95,108].

*ESSRG* was found in the Brazilian buffalo breed by both buffalo [111] and bovine [108] SNP chips. Both in Italian Mediterranean [113,115] and Brazilian buffalo breeds [108], *APOB* was identified by different SNP chips. The interactive relationship of the *APOB* was observed with candidate genes, including thyroglobulin (*TG*), leptin (*LEP*), and sterol regulatory element-binding transcription factor 1 (*SREBF1*) [107]. Low-density lipoproteins and chylomicrons apolipoprotein are the main products of the *APOB* gene. Recently, Lee et al. [116] reported that *APOB* is involved in the regulation of lipid metabolism.

In river buffaloes, a non-synonymous mutation in exon 10 of the insulin-like growth factor 2 (*IGF2*) gene has shown a potential association with higher daily weight gain from birth to 36 weeks [117]. Furthermore, in Mediterranean river buffalo females, the C > T substitution in *STAT5A* was associated with milk protein percentage [118]. El-Komy et al. [119] screened the *GHR* polymorphisms and their potential association with milk yield traits in Egyptian buffaloes (400 animals). The mutations in four exons (E4–E6 and E8) of the *GHR* gene were investigated, and in E4 no variation was detected, while two SNPs in E5 (c.380G > A/p.Arg127Lys and c.387C > T/p.Gly129), a single silent alteration (c.435A > G/p.Pro145) in E6, and an additional missense mutation (c.836T > A/p.Phe279Tyr) in E8 were spotted. Two SNPs c.380G > A and c.836T > A in the extracellular and transmembrane regions, respectively, were related with milk yield; protein percentage; fat percentage; and 305-day milk, protein, and fat yield, with higher levels in individuals possessing the mutant A allele. Remarkably, the animal with two mutant alleles (AA) gave a higher milk yield, with higher protein and fat percentages, by upregulating the expression of *GH*, *GHR*, prolactin (*PRL*), prolactin receptor (*PRLR*), diacylglycerol acyltransferase-1 (*DGAT1*), *CSN2* gene-encoded beta-casein and insulin-like growth factor 1 (*IGF1*) genes, and proteins in milk-producing cells [119]. In the *CSN1S2* gene, the coding sequence of river and swamp buffaloes, 13 SNPs were identified, including 8 non-synonymous substitutions. The amino acid variations due to c.580T > C and c.642C > G might have a physiological impact on the αS2-CN synthesis and ultimately the milk yield in buffaloes [120,121].

Recent genome-wide studies have detected 8 *DGAT* genes in buffaloes, which were grouped into two subfamilies, *DGAT1* and *DGAT2*, in comparison to 15 *DGAT* homologous proteins in *Bos taurus* [122]. Association of *DGAT* genes with milk production traits was analyzed by using data from 489 buffaloes with 1424 lactations [122], and 20 SNPs associated with different milk production traits were identified. The most significant SNP in the *DGAT1* genomic region (Affx-79,549,398) was associated with buffalo milk protein and fat percentages.

#### 5.1.4. Body Coat Color

Visible skin-color phenotypes can be used to discover gene expression regulation and the patterns of coat color evolution in animals [123]. The dark-gray body color is common in swamp buffaloes, while in some populations, the white coat color variant is prevalent up to 10% [124]. In swamp buffaloes, genetic analysis revealed the dominance of white color over black [125]. Missense mutations in the *MC1R* gene of river buffaloes were identified, but these variations did not explain the difference between the dark-gray and white color of swamp buffaloes [126]. In Indonesia (Tana Toraja), mainly the white-spotted buffalo bulls are highly prized and slaughtered in funeral ceremonies [127]. In microphthalmia-associated transcription factor (*MITF*), two independent mutations that caused functional loss, a donor splice-site alteration and a premature stop codon, were importantly linked with white-spotted skin color [127].

Liang et al. [123] conducted a whole-genome-sequencing-based study to map the swamp buffalo white coat phenotype. The comparative population-based genomic analyses (41 black and 22 white swamp buffaloes) detected the signatures of the selection underlying the white coat phenotype. In the agouti signaling protein (*ASIP*) gene, Liang et al. [124] reported in LINE-1 a 2809-bp-long insertion, which is the reason for the white coat pigmentation in swamp buffaloes. In the white body coat, the LINE-1 insertion acted as a potential proximal promoter, which increased the transcription of *ASIP* 10-fold. The transcribed 165 bp of 5′ UTR from LINE-1 is combined with *ASIP* first coding exon, and a chimeric transcript is developed. The enhanced expression of *ASIP* prevents the maturation of melanocytes, resulting in the absence of color in white buffaloes’ hairs and skin. The phylogenetic analyses specified recent genetic transposition events that evolve a specific *ASIP* allele related to buffalo white coloration in swamp buffaloes. Moreover, in cattle *ASIP* gene, similar insertion of LINE-1 has been identified, which is evident for the convergent mechanism of evolution for coat color in the Bovini tribe [123].

#### 5.1.5. Disease Resistance

Water buffaloes’ susceptibility or resistance to a specific disease, like tuberculosis or mastitis, has also been influenced by some genetic variants. The higher vulnerability of Mediterranean buffaloes to bovine tuberculosis was associated with an SNP (G > A) at position 4467 in the 3′ UTR of the interferon-gamma (*IFNG*) gene, which caused the target sequence disruption for the micro-RNA (miR-125b) [128]. A substitution C > A in exon 27 of the *C3* (complement component 3) gene was traced in the buffalo population that is significantly associated with the somatic cell score, which is a potential indicator of mastitis in dairy animals [129].

Globally, in animals, foot-and-mouth disease (FMD) is caused by single-stranded RNA virus (FMDV) infection, which leads to economic losses in livestock production. The resistance or susceptibility of FMD in buffaloes is owing to genetic variations in the *BoLA-DRB3* gene. In exon 2 of the *BoLA-DRB3* gene, 302-bp-amplified fragments were digested with *HaeIII* endonuclease enzyme and five *BoLA-DRB3* genotypes were traced and the genotype AA was correlated with FMD resistance. However, the AC genotype might be associated with FMD susceptibility in Egyptian buffaloes [130]. Furthermore, in cattle, the natural resistance to brucellosis has been associated with the (GT)13 microsatellite allele of *SLC11A1* at 3′ UTR [131]. Various other reports have also discovered a strong association of (GT)n microsatellite alterations with vulnerability to or resistance against brucellosis in buffaloes [131,132,133,134].

## 6. The Future Perspective of Buffaloes

Domesticated water buffaloes are a proficient source of nutritious products, particularly milk and meat, and have an excellent capability to live on marginal resources under adverse environmental conditions [5,135]. Buffaloes are an important dairy species due to the peculiar taste of their milk and unique milk products, like Mozzarella cheese, in addition to their socio-cultural significance. Owing to better climate resilience and adaptability attributes, buffaloes would be important in the future climate change scenario for sustainable dairy production setups where *Bos taurus* cannot thrive or perform well, particularly in tropical areas.

To exploit the excellent potential of buffaloes in terms of superior production performance and climate resilience, it is imperative to accelerate genetic progress in this species by using genomic selection and improved breeding. Genomic selection has proven successful in cattle for making genetic improvements and increasing the rates of genetic gain and is equally feasible for buffalo improvement [136]. A simulated study has shown that using genomic selection in buffaloes can reduce the generation interval from 9.5 to 3.3 years (for male path) while increasing selection response by two times and reducing the cost of proving bulls by 88% [137]. This envisages future advancement in water buffalo breeding programs by exploiting genomic selection. No doubt the use of genomic information in genetic evaluation of buffaloes is still in its infancy, but it has a very bright future as remarkable progress is expected in buffalo breeding using genomic information. The advancement in the field of genomics will also facilitate a better understanding of the unique genetic features of water buffaloes and will also provide more influential tools for the genome-based identification of quantitative trait loci (QTL) and candidate genes. This would help to understand the regulatory mechanism of fundamental traits, including adaptation, disease resistance, and production.

Further, genome editing technologies are also likely to enhance the genetic potential of buffalo populations. So far, few studies involving the whole buffalo genome have been documented; therefore, buffalo genomic data are still scanty. Thus, there is a dire need to study the genomic annotation of buffaloes on a wider scale using high-throughput technologies. Furthermore, the identification of functional candidate genes should be given more consideration to explore the genetic variants associated with production performance, climate adaptation, and disease resistance. Taken together, genome-wide information would facilitate future initiatives focusing on genetic improvement and effective use of buffalo genetic resources globally.

## 7. Conclusions

Recent advancements in high-throughput technologies like whole-genome sequencing, GWAS, gene expression profiling, next-generation sequencing (RNA and DNA), and genome-wide CHIP-seq scanning are used to detect the genetic variants, elucidate gene regulation, and perform function profiling in buffaloes. These offer wide-ranging whole-genome data and high coverage to genomic, epigenomic, transcriptomic, and proteomic information related to cellular interactions, functioning, and behavior. In buffaloes, candidate gene studies have used the available genetic resources to uncover the functional candidate genes and their potential association with buffalo performance, including production, adaptation, and disease resistance. Thus, the whole-genome and candidate gene approach to next-generation data could be helpful in elucidating the complex traits, genomic coverage, and productivity-related attributes, which will facilitate improved breeding and better use of buffalo genetic resources.

## Figures and Tables

**Table 1:** Genome sequences statistics, annotation, and population parameters.

		Swamp Buffalo	River Buffalo	Mediterranean Buffalo
**Genome**	Total genome size (Mb)	2631	2646	2654
	Chromosome number	48	50	50
	Scaffold number	24 + 1510	25 + 2279	25 + 506
	Scaffold N50 (Mb)	117.3	116.1	117.2
	Total contig size (Mb)	2609	2626	2622
	Contig N50 (Mb)	8.8	3.1	18.8
**Annotation**	Total genes	19,272	20,202	24,014
	Average CDS length (bp)	1764.5	1662.2	-
	BUSCO assessment	96.80%	96.00%	93.6%
	Repeat content	47.26%	47.31	45.89%
**Population**	Sample number	132	98	-
	Number of SNPs	18,737,564	23,722,820	-
	Genetic diversity θ (4 Nμ)	0.001805	0.002743	-
	Population differentiation (Fst)	0.27		-

**Table 2:** Candidate genes associated with different milk yield traits.

No.	Trait	Candidate Genes
1	Milk yield	*STAT1*, *STAT5A*, *LEP*, *MC4R*, *OXT*, *INSIG2*, *LALBA*, *BTN1A1*, *PRL*, *SCD*, and *SREBF1*
2	Milk fat yield	*GHRL* and *A2M*
3	Milk fat (%)	*STAT1*, *TG*, *A2M*, *DGAT1*, *GHRL*, *LEP*, *MC4R*, *PRL*, *SCD*, and *SREBF1*
4	Milk protein (%)	*CSN1S1*, *DGAT1*, *GHRL*, *ADRA1A*, *A2M MTNR1A*, *PRL* and *SPP1*, *INSIG2*, and *MC4R*

## Data Availability

Not applicable.

## References

[1] Kierstein G., Vallinoto M., Silva A., Schneider M.P., Iannuzzi L., Brenig B. (2004). Analysis of mitochondrial D-loop region casts new light on domestic water buffalo (*Bubalus bubalis*) phylogeny. Mol. Phylogenet. Evol..

[2] Roth J., Myers P. Bubalus Bubalis 2004. http://animaldiversity.ummz.umich.edu/site/accounts/information/Bubalus_bubalis.html.

[3] Yindee M., Vlamings B.H., Wajjwalku W., Techakumphu M., Lohachit C., Sirivaidyapong S., Thitaram C., Amarasinghe A.A., Alexander P.A., Colenbrander B. (2010). Y-chromosomal variation confirms independent domestications of swamp and river buffalo. Anim. Genet..

[4] (2019). FAO. http://www.fao.org/faostat/en/#data/QA/visualize.

[5] Rehman S., Shafique L., Yousuf M.R., Liu Q., Ahmed J.Z., Riaz H. (2019). Spectrophotometric calibration and comparison of different semen evaluation methods in Nili-Ravi buffalo bulls. Pak. Vet. J..

[6] Council N.R. (1981). The Water Buffalo: New Prospects for an Underutilized Animal.

[7] Akhtar M.S., Lodhi L.A., Ahmad I., Qureshi Z.I., Muhammad G. (2012). Serum trace mineral variations in Nili-Ravi buffaloes suffering with prepartum vaginal prolapse in two different agro-ecological zones of Punjab, Pakistan. Theriogenology.

[8] Warriach H.M., McGill D.M., Bush R.D., Wynn P.C., Chohan K.R. (2015). A review of recent developments in buffalo reproduction—A review. Asian-Australas. J. Anim. Sci..

[9] Haldar A., Prakash B.S. (2005). Peripheral patterns of growth hormone, luteinizing hormone, and progesterone before, at, and after puberty in buffalo heifer. Endocr. Res..

[10] De Rensis F., Lopez-Gatius F. (2007). Protocols for synchronizing estrus and ovulation in buffalo (Bubalus bubalis): A review. Theriogenology.

[11] Hassan F., Khan M.S., Rehman M.S., Sarwar M., Bhatti S.A. (2007). Seasonality of calving in Nili-Ravi buffaloes, purebred Sahiwal and crossbred cattle in Pakistan. Ital. J. Anim. Sci..

[12] Perera B.M.A.O. (2011). Reproductive cycles of buffalo. Anim. Reprod. Sci..

[13] Hussain Shah S.N. (2007). Prolonged calving intervals in the Nili ravi buffalo. Ital. J. Anim. Sci..

[14] El-Wishy A.B. (2007). The postpartum buffalo: II. Acyclicity and anestrus. Anim. Reprod. Sci..

[15] Fahimuddin M. (1975). Domestic water buffalo. Domestic Water Buffalo.

[16] Berardino D.D., Iannuzzi L. (1981). Chromosome banding homologies in Swamp and Murrah buffalo. J. Hered..

[17] Iannuzzi L., Di Meo G., Cockett N.E., Kole C. (2009). Water Buffalo. Genome Mapping and Genomics in Domestic Animals.

[18] De Abreu Santos D.J., Ferreira de Camargo G.M., Cardoso D.F., Buzanskas M.E., Aspilcueta-Borquis R.R., Hurtado-Lugo N.A., de Araújo Neto F.R., Galvão de Albuquerque L., Ma L., Tonhati H. (2020). Linkage disequilibrium-based inference of genome homology and chromosomal rearrangements between species. G3 Genes Genomes Genet..

[19] Amaral M.E., Grant J.R., Riggs P.K., Stafuzza N.B., Rodrigues Filho E.A., Goldammer T., Weikard R., Brunner R.M., Kochan K.J., Greco A.J. (2008). A first-generation whole genome RH map of the river buffalo with comparison to domestic cattle. BMC Genom..

[20] Amaral M.E., Owens K.E., Elliott J.S., Fickey C., Schäffer A.A., Agarwala R., Womack J.E. (2007). Construction of a river buffalo (*Bubalus bubalis*) whole-genome radiation hybrid panel and preliminary RH mapping of chromosomes 3 and 10. Ital. J. Anim. Sci..

[21] Luo X., Zhou Y., Zhang B., Zhang Y., Wang X., Feng T., Li Z., Cui K., Wang Z., Luo C. (2020). Understanding divergent domestication traits from the whole-genome sequencing of swamp-and river-buffalo populations. Natl. Sci. Rev..

[22] Low W.Y., Tearle R., Bickhart D.M., Rosen B.D., Kingan S.B., Swale T., Thibaud-Nissen F., Murphy T.D., Young R., Lefevre L. (2019). Chromosome-level assembly of the water buffalo genome surpasses human and goat genomes in sequence contiguity. Nat. Commun..

[23] Gallagher D.S., Womack J.E. (1992). Chromosome conservation in the Bovidae. J. Hered..

[24] Iannuzzi L., Di Meo G.P., Perucatti A., Schibler L., Incarnato D., Cribiu E.P. (2001). Comparative FISH mapping in river buffalo and sheep chromosomes: Assignment of forty autosomal type I loci from sixteen human chromosomes. Cytogenet. Genome Res..

[25] Iannuzzi L., King W.A., Di Berardino D. (2009). Chromosome evolution in domestic bovids as revealed by chromosome banding and FISH-mapping techniques. Cytogenet. Genome Res..

[26] Amano T., Miyakoshi Y., Takada T., Kikkawa Y., Suzuki H. (1994). Genetic variants of ribosomal DNA and mitochondrial DNA between swamp and river buffaloes. Anim. Genet..

[27] Tanaka K., Solis C.D., Masangkay J.S., Maeda K., Kawamoto Y., Namikawa T. (1996). Phylogenetic relationship among all living species of the genus Bubalus based on DNA sequences of the cytochrome b gene. Biochem. Genet..

[28] Barker J.S., Tan S.G., Selvaraj O.S., Mukherjee T.K. (1997). Genetic variation within and relationships among populations of Asian water buffalo (Bubalus bubalis). Anim. Genet..

[29] Barker J.S., Moore S.S., Hetzel D.J., Evans D., Byrne K., Tan S.G. (1997). Genetic diversity of Asian water buffalo (Bubalus bubalis): Microsatellite variation and a comparison with protein-coding loci. Anim. Genet..

[30] Lau C.H., Drinkwater R.D., Yusoff K., Tan S.G., Hetzel D.J.S., Barker J.S.F. (1998). Genetic diversity of Asian water buffalo (Bubalus bubalis): Mitochondrial DNA D-loop and cytochrome b sequence variation. Anim. Genet..

[31] Lei C.Z., Zhang W., Chen H., Lu F., Liu R.Y., Yang X.Y., Zhang H.C., Liu Z.G., Yao L.B., Lu Z.F. (2007). Independent maternal origin of Chinese swamp buffalo (*Bubalus bubalis*). Anim. Genet..

[32] Kumar S., Nagarajan M., Sandhu J.S., Kumar N., Behl V., Nishanth G. (2007). Mitochondrial DNA analyses of Indian water buffalo support a distinct genetic origin of river and swamp buffalo. Anim. Genet..

[33] Mishra B.P., Dubey P.K., Prakash B., Kathiravan P., Goyal S., Sadana D.K., Das G.C., Goswami R.N., Bhasin V., Joshi B.K. (2015). Genetic analysis of river, swamp and hybrid buffaloes of north-east India throw new light on phylogeography of water buffalo (*Bubalus bubalis*). J. Anim. Breed. Genet..

[34] Wang S., Chen N., Capodiferro M.R., Zhang T., Lancioni H., Zhang H., Miao Y., Chanthakhoun V., Wanapat M., Yindee M. (2017). Whole mitogenomes reveal the history of swamp buffalo: Initially shaped by glacial periods and eventually modelled by domestication. Sci. Rep..

[35] Takahata N., Nei M. (1985). Gene genealogy and variance of interpopulational nucleotide differences. Genetics.

[36] Nagarajan M., Nimisha K., Kumar S. (2015). Mitochondrial DNAvariability of domestic river buffalo (*Bubalus bubalis*) populations: Genetic evidence for domestication of river buffalo in Indian subcontinent. Genome Biol. Evol..

[37] Colli L., Milanesi M., Vajana E., Iamartino D., Bomba L., Puglisi F., Del Corvo M., Nicolazzi E.L., Ahmed S.S., Herrera J.R. (2018). New insights on water buffalo genomic diversity and post-domestication migration routes from medium density SNP chip data. Front. Genet..

[38] Kumar S., Nagarajan M., Sandhu J.S., Kumar N., Behl V. (2007). Phylogeography and domestication of Indian river buffalo. BMC Evol. Biol..

[39] Zhang Y., Vankan D., Zhang Y., Barker J.S. (2011). Genetic differentiation of water buffalo (*Bubalus bubalis*) populations in China, Nepal and south-east Asia: Inferences on the region of domestication of the swamp buffalo. Anim. Genet..

[40] Zhang Y., Lu Y., Yindee M., Li K.Y., Kuo H.Y., Ju Y.T., Ye S., Faruque M.O., Li Q., Wang Y. (2016). Strong and stable geographic differentiation of swamp buffalo maternal and paternal lineages indicates domestication in the China/Indochina border region. Mol. Ecol..

[41] Finlay E.K., Gaillard C., Vahidi S.M., Mirhoseini S.Z., Jianlin H., Qi X.B., El-Barody M.A., Baird J.F., Healy B.C., Bradley D.G. (2007). Bayesian inference of population expansions in domestic bovines. Biol. Lett..

[42] Clutton-Brock J. (1999). A natural History of Domesticated Mammals.

[43] Zeuner F.E. (1963). A History of Domesticated Animals.

[44] Unal E.O., Soysal M.I., Yuncu E., Dagtas N.D., Togan I. (2014). Microsatellite based genetic diversity among the three-water buffalo (*Bubalus bubalis*) populations in Turkey. Arch. Anim. Breed..

[45] Zhang Y., Colli L., Barker J.S. (2020). Asian water buffalo: Domestication, history and genetics. Anim. Genet..

[46] Higham C. (2002). Early Cultures of Mainland Southeast Asia.

[47] Sun T., Shen J., Achilli A., Chen N., Chen Q., Dang R., Zhang T. (2020). Genomic analyses reveal distinct genetic architectures and selective pressures in buffaloes. GigaScience.

[48] Larson G., Burger J. (2013). A population genetics view of animal domestication. Trends Genet.

[49] NCBI. https://www.ncbi.nlm.nih.gov.

[50] Mintoo A.A., Zhang H., Chen C., Moniruzzaman M., Deng T., Anam M., Han P. (2019). Draft genome of the river water buffalo. Ecol. Evol..

[51] Kent W.J. (2002). BLAT–the BLAST-like alignment tool. Genome Res..

[52] Han L., Su B., Li W.H., Zhao Z. (2008). CpG island density and its correlations with genomic features in mammalian genomes. Genome Biol..

[53] Jobse C., Buntjer J.B., Haagsma N., Breukelman H.J., Beintema J.J., Lenstral J.A. (1995). Evolution and recombination of bovine DNA repeats. J. Mol. Evol..

[54] Elsik C.G., Tellam R.L., Worley K.C. (2009). The genome sequence of taurine cattle: A window to ruminant biology and evolution. Science.

[55] Li J., Liu J., Campanile G., Plastow G., Zhang C., Wang Z., Yang L. (2018). Novel insights into the genetic basis of buffalo reproductive performance. BMC Genom..

[56] Glanzmann B., Möller M., Le Roex N., Tromp G., Hoal E.G., Van Helden P.D. (2016). The complete genome sequence of the African buffalo (*Syncerus caffer*). BMC Genom..

[57] Dutta P., Talenti A., Young R., Jayaraman S., Callaby R., Jadhav S.K., Dhanikachalam V., Manikandan M., Biswa B.B., Low W.Y. (2020). Whole genome analysis of water buffalo and global cattle breeds highlights convergent signatures of domestication. Nat. Commun..

[58] Mishra S.R. (2020). Significance of molecular chaperones and micro RNAs in acquisition of thermo-tolerance in dairy cattle. Anim. Biotechnol..

[59] Rehman S., Nadeem A., Javed M., Hassan F., Luo X., Khalid R.B., Liu Q. (2020). Genomic Identification, Evolution and Sequence Analysis of the Heat-Shock Protein Gene Family in Buffalo. Genes.

[60] Archana P.R., Aleena J., Pragna P., Vidya M.K., Niyas A.P., Bagath M., Krishnan G., Manimaran A., Beena V., Kurien E.K. (2017). Role of heat shock proteins in livestock adaptation to heat stress. J. Dairy Vet. Anim. Res..

[61] Collier R.J., Collier J.L., Rhoads R.P., Baumgard L. (2008). Invited review: Genes involved in the bovine heat stress response. J. Dairy Sci..

[62] Mishra A., Hooda O.K., Singh G., Meur S.K. (2011). Influence of induced heat stress on HSP70 in buffalo lymphocytes. J. Anim. Physiol. Anim. Nutr..

[63] Cushman R.A. (2013). Physiology and endocrinology symposium: The current status of heat shock in early embryonic survival and reproductive efficiency. J. Anim. Sci..

[64] Dong C.W., Zhang Y.B., Zhang Q.Y., Gui J.F. (2006). Differential expression of three Paralichthys olivaceus Hsp40 genes in responses to virus infection and heat shock. Fish Shellfish Immunol..

[65] Banerji U. (2009). Heat Shock Protein 90 as a Drug Target: Some Like It Hot. Clin. Cancer Res..

[66] Chen W., Feng P., Liu T., Jin D. (2019). Recent advances in machine learning methods for predicting heat shock proteins. Curr. Drug Metab..

[67] Liu Y.X., Li D.Q., Cui Q.W., Shi H.X., Wang G.L. (2010). Analysis of HSP70 mRNA level and association between linked microsatellite loci and heat tolerance traits in dairy cows. Yi Chuan Hered..

[68] Hassan F., Nawaz A., Rehman M.S., Ali M.A., Dilshad S.M., Yang C. (2019). Prospects of HSP70 as a genetic marker for thermo-tolerance and immuno-modulation in animals under climate change scenario. Anim. Nutr..

[69] Sodhi M., Mukesh M., Kishore A., Mishra B., Kataria R., Joshi B. (2013). Novel polymorphisms in UTR and coding region of inducible heat shock protein 70.1 gene in tropically adapted Indian zebu cattle (*Bos indicus*) and riverine buffalo (*Bubalus bubalis*). Gene.

[70] Rosenkrans C., Banks A., Reiter S., Looper M. (2010). Calving traits of crossbred Brahman cows are associated with Heat Shock Protein 70 genetic polymorphisms. Anim. Reprod. Sci..

[71] Barile V.L. (2005). Improving reproductive efficiency in female buffaloes. Livest. Prod. Sci..

[72] Shi W., Yuan X., Cui K., Li H., Fu P., Rehman S.U., Shi D., Liu Q., Li Z. (2021). LC-MS/MS Based Metabolomics Reveal Candidate Biomarkers and Metabolic Changes in Different Buffalo Species. Animals.

[73] Cheng Y., Gu J., Xue H., Li Q., Liang M., Wang N., Wang G., Wu Q., Liu S., Yu H. (2017). Identification of four SNPs in LHB gene and their associations with sperm qualities of Chinese buffaloes. Anim. Biotechnol..

[74] Wang G., Hao L., Cheng Y., Li S., Zhang Y., Lv C., Wei W., Huang S., Shi H., Dong L. (2017). Effects of GnRHR polymorphisms on sperm quality in Chinese water buffalo. Anim. Reprod. Sci..

[75] El-Bayomi K.M., Saleh A.A., Awad A., El-Tarabany M.S., El-Qaliouby H.S., Afifi M., El-Komy S., Essawi W.M., Almadaly E.A., El-Magd M.A. (2018). Association of CYP19A1 gene polymorphisms with anoestrus in water buffaloes. Reprod. Fertil. Dev..

[76] Pandey A.K., Gunwant P., Soni N., Kumar S., Kumar A., Magotra A., Singh I., Phogat J.B., Sharma R.K., Bangar Y. (2019). Genotype of MTNR1A gene regulates the conception rate following melatonin treatment in water buffalo. Theriogenology.

[77] Surya T., Vineeth M.R., Sivalingam J., Tantia M.S., Dixit S.P., Niranjan S.K., Gupta I.D. (2019). Genomewide identification and annotation of SNPs in Bubalus bubalis. Genomics.

[78] Chethan S.G., Singh S.K., Nongsiej J., Rakesh H.B., Singh R.P., Kumar N., Agarwal S.K. (2014). IFN-τ Acts in a Dose-Dependent Manner on Prostaglandin Production by Buffalo Endometrial Stromal Cells Cultured in vitro. Reprod. Domest. Anim..

[79] Fortes M.R.S., Lehnert S.A., Bolormaa S., Reich C., Fordyce G., Corbet N.J., Whan V., Hawken R.J., Reverter A. (2012). Finding genes for economically important traits: Brahman cattle puberty. Anim. Prod Sci..

[80] McDaneld T.G., Kuehn L.A., Thomas M.G., Snelling W.M., Smith T.P.L., Pollak E.J., Cole J.B., Keele J.W. (2014). Genomewide association study of reproductive efficiency in female cattle. J. Anim. Sci..

[81] De Camargo G.M., Porto-Neto L.R., Kelly M.J., Bunch R.J., McWilliam S.M., Tonhati H., Lehnert S.A., Fortes M.R., Moore S.S. (2015). Non-synonymous mutations mapped to chromosome X associated with andrological and growth traits in beef cattle. BMC Genom..

[82] Arndt L., Castonguay J., Arlt E., Meyer D., Hassan S., Borth H., Zierler S., Wennemuth G., Breit A., Biel M. (2014). NAADP and the two-pore channel protein 1 participate in the acrosome reaction in mammalian spermatozoa. Mol. Biol. Cell.

[83] Spencer T.E., Forde N., Dorniak P., Hansen T.R., Romero J.J., Lonergan P. (2013). Conceptus-derived prostaglandins regulate gene expression in the endometrium prior to pregnancy recognition in ruminants. Reproduction.

[84] Seo H., Choi Y., Shim J., Yoo I., Ka H. (2014). Prostaglandin transporters ABCC4 and SLCO2A1 in the uterine endometrium and conceptus during pregnancy in pigs. Biol. Reprod..

[85] Refsdal A.O. (2000). To treat or not to treat: A proper use of hormones and antibiotics. Anim. Reprod. Sci..

[86] Khaitan D., Sankpal U.T., Weksler B., Meister E.A., Romero I.A., Couraud P.O., Ningaraj S.N. (2009). Role of KCNMA1 gene in breast cancer invasion and metastasis to brain. BMC Cancer.

[87] Peng L., Zhang S.D., Yuen H.F., Mccrudden C.M., Wen Q., Chan K.W., Hang F.K. (2017). Identification of TWIST-interacting genes in prostate cancer. Sci. China Life Sci..

[88] An C.H., Je E.M., Yoo N.J., Lee S.H. (2015). Frameshift mutations of cadherin genes DCHS2, CDH10 and CDH24 genes in gastric and colorectal cancers with high microsatellite instability. Pathol. Oncol. Res..

[89] Işık E., Beck P.P., Campi I., Özon A., Alikaşifoğlu A., Gönç N., Kandemir N. (2013). Thyroid hormone resistance: A novel mutation in thyroid hormone receptor beta (THRB) gene—Case report. Turk. J. Pediatr..

[90] Barrett P., Ebling F.J., Schuhler S., Wilson D., Ross A.W., Warner A., Jethwa P., Boelen A., Visser T.J., Ozanne D.M. (2007). Hypothalamic thyroid hormone catabolism acts as a gatekeeper for the seasonal control of body weight and reproduction. Endocrinology.

[91] Rodriguez-Castelãn J., Anaya-Hernãndez A., Mendez-Tepepa M., Martinez-Gomez M., Castelãn F., Cuevas-Romero E. (2016). Distribution of thyroid hormoneand thyrotropin receptors in reproductive tissues of adult female rabbits. Endocr. Res. Commun..

[92] Christ G.J., Andersson K.E., Williams K., Zhao W., D’Agostino R., Kaplan J., Aboushwareb T., Yoo J., Calenda G., Davies K.P. (2009). Smooth-muscle–specific gene transfer with the human Maxi-K channel improves erectile function and enhances sexual behavior in atherosclerotic cynomolgus monkeys. Eur. Urol..

[93] Gibbons J.R., Wiltbank M.C., Ginther O.J. (1999). Relationship between follicular development and the decline in the follicle-stimulating hormone surge in heifers. Biol. Reprod..

[94] Li W., Bickhart D.M., Ramunno L., Iamartino D., Williams J.L., Liu G.E. (2018). Comparative sequence alignment reveals River Buffalo genomic structural differences compared with cattle. Genomics.

[95] Wu J.J., Song L.J., Wu F.J., Liang X.W., Yang B.Z., Wathes D.C., Pollott G.E., Cheng Z., Shi D.S., Liu Q.Y. (2013). Investigation of transferability of BovineSNP50 BeadChip from cattle to water buffalo for genome wide association study. Mol. Biol. Rep..

[96] Shahi P., Slorach E.M., Wang C.Y., Chou J., Lu A., Ruderisch A., Werb Z. (2015). The transcriptional repressor ZNF503/Zeppo2 promotes mammary epithelial cell proliferation and enhances cell invasion. J. Biol. Chem..

[97] Chang S.L., Liu Y.C., Chen S.Y., Huang T.H., Liu P.T., Liu F.C. (2013). Identification of two evolutionarily conserved 5′cis-elements involved in regulating spatiotemporal expression of Nolz-1 during mouse embryogenesis. PLoS ONE.

[98] Day F.R., Hinds D.A., Tung J.Y., Stolk L., Styrkarsdottir U., Saxena R., Bjonnes A., Broer L., Dunger D.B., Halldorsson B.V. (2015). Causal mechanisms and balancing selection inferred from genetic associations with polycystic ovary syndrome. Nat. Commun..

[99] Makina S.O., Muchadeyi F.C., van Marle-Koster E., Taylor J.F., Makgahlela M.L., Maiwashe A. (2015). Genome-wide scan for selection signatures in six cattle breeds in South Africa. Genet. Sel. Evol..

[100] Wang L., Wang Y. (2012). Molecular characterization, expression patterns and subcellular localization of Myotrophin (MTPN) gene in porcine skeletal muscle. Mol. Biol. Rep..

[101] Kato M.V. (2000). A secreted tumor-suppressor, mac25, with activin-binding activity. Mol. Med..

[102] Ueno N., Ling N., Ying S.Y., Esch F., Shimasaki S., Guillemin R. (1987). Isolation and partial characterization of follistatin: A single-chain Mr 35,000 monomeric protein that inhibits the release of follicle-stimulating hormone. Proc. Natl. Acad. Sci. USA.

[103] Robertson D.M., Klein R., de Vos F.L., Mclachlan R.I., Wettenhall R.E., Hearn M.T., Burger H.G., de Kretser D.M. (1987). The isolation of polypeptides with FSH suppressing activity from bovine follicular fluid which are structurally different to inhibin. Biochem. Biophys. Res. Commun..

[104] Jorgez C.J., Klysik M., Jamin S.P., Behringer R.R., Matzuk M.M. (2004). Granulosa cellspecific inactivation of follistatin causes female fertility defects. Mol. Endocrinol..

[105] Muttukrishna S., Tannetta D., Groome N., Sargent I. (2004). Activin and follistatin in female reproduction. Mol. Cell. Endocrinol..

[106] Okamura H., Yamamura T., Wakabayashi Y. (2013). Kisspeptin as a master player in the central control of reproduction in mammals: An overview of kisspeptin research in domestic animals. Anim. Sci. J..

[107] Du C., Deng T., Zhou Y., Ye T., Zhou Z., Zhang S., Shao B., Wei P., Sun H., Khan F.A. (2019). Systematic analyses for candidate genes of milk production traits in water buffalo (*Bubalus Bubalis*). Anim. Genet..

[108] Venturini G.C., Cardoso D.F., Baldi F., Freitas A.C., Aspilcuetaborquis R.R., Santos D.J., Camargo G.M., Stafuzza N.B., Albuquerque L.G., Tonhati H. (2014). Association between single-nucleotide polymorphisms and milk production traits in buffalo. Genet. Mol. Res..

[109] Michelizzi V.N., Dodson M.V., Pan Z., Amaral M.E.J., Michal J.J., Mclean D.J., Womack J.E., Jiang Z. (2010). Water buffalo genome science comes of age. Int. J. Biol. Sci..

[110] Cesarani A., Gaspa G., Pauciullo A., Degano L., Vicario D., Macciotta N.P. (2021). Genome-wide analysis of homozygosity regions in european simmental bulls. J. Anim. Breed. Genet..

[111] Camargo G.D., Aspilcuetaborquis R.R., Fortes M., Portoneto R., Cardoso D.F., Santos D., Lehnert S.A., Reverter A., Moore S.S., Tonhati H. (2015). Prospecting major genes in dairy buffaloes. BMC Genom..

[112] El-Halawany N., Abdel-Shafy H., Shawky A.E.M.A., Abdel-Latif M.A., Al-Tohamy A.F.M., El-Moneim O.M.A. (2017). Genomewide association study for milk production in Egyptian buffalo. Livest. Sci..

[113] Iamartino D., Nicolazzi E.L., Van C.T., Reecy J.M., Fritzwaters E.R., Koltes J.E., Biffani S., Sonstegard T.S., Schroeder S.G., Ajmonemarsan P. (2017). Design and validation of a 90K SNP genotyping assay for the water buffalo (*Bubalus bubalis*). PLoS ONE.

[114] Liu J.J., Liang A.X., Campanile G., Plastow G., Zhang C., Wang Z., Salzano A., Gasparrini B., Cassandro M., Yang L.G. (2017). Genome-wide association studies to identify quantitative trait loci affecting milk production traits in water buffalo. J. Dairy Sci..

[115] Gu M., Cosenza G., Gaspa G., Iannaccone M., Macciotta N.P., Chemello G., Di Stasio L., Pauciullo A. (2020). Sequencing of lipoprotein lipase gene in the Mediterranean river buffalo identified novel variants affecting gene expression. J. Dairy Sci..

[116] Lee S., Bao H., Ishikawa Z., Wang W., Lim H.Y. (2017). Cardiomyocyte regulation of systemic lipid metabolism by the apolipoprotein B-containing lipoproteins in drosophila. PLoS Genet..

[117] Abo-Al-Ela H.G., El-Magd M.A., El-Nahas A.F., Mansour A.A. (2014). Association of a novel SNP in exon 10 of the IGF2 gene with growth traits in Egyptian water buffalo (*Bubalus bubalis*). Trop. Anim. Health Prod..

[118] Coizet B., Frattini S., Nicoloso L., Iannuzzi L., Coletta A., Talenti A., Minozzi G., Pagnacco G., Crepaldi P. (2018). Polymorphism of the STAT5A, MTNR1A and TNFα genes and their effect on dairy production in Bubalus bubalis. Ital. J. Anim. Sci..

[119] El-Komy S.M., Saleh A.A., Abdel-Hamid T.M., El-Magd M.A. (2020). Association of GHR Polymorphisms with Milk Production in Buffaloes. Animals.

[120] Fan X., Gao S., Fu L., Qiu L., Miao Y. (2020). Polymorphism and molecular characteristics of the CSN1S2 gene in river and swamp buffalo. Arch. Anim. Breed..

[121] Rehman S.U., Feng T., Wu S., Luo X., Lei A., Luobu B., Hassan F., Liu Q. (2021). Comparative genomics, evolutionary and gene regulatory regions analysis of casein gene family in *Bubalus bubalis*. Front. Genet..

[122] Liu J., Wang Z., Li J., Li H., Yang L. (2020). Genome-wide identification of Diacylglycerol Acyltransferases (DGAT) family genes influencing Milk production in Buffalo. BMC Genet..

[123] Liang D., Zhao P., Si J., Fang L., Pairo-Castineira E., Hu X., Tian B. (2020). Genomic analysis revealed a convergent evolution of LINE-1 in coat color: A case study in water buffaloes (*Bubalus bubalis*). Mol. Biol. Evol..

[124] Rife D.C. (1962). Color and horn variations in water buffalo: The inheritance of coat color, eye color and shape of horns. J. Hered..

[125] Rife D.C., Buranamanas P. (1959). Inheritance of white coat color in the water buffalo of Thailand. J. Hered..

[126] Miao Y., Wu G., Wang L., Li D., Tang S., Liang J., Mao H., Luo H., Zhang Y. (2010). The role of MC1R gene in buffalo coat color. Sci. China Life Sci..

[127] Yusnizar Y., Wilbe M., Herlino A.O., Sumantri C., Noor R.R., Boediono A., Andersson L., Andersson G. (2015). Microphthalmia-associated transcription factor mutations are associated with white-spotted coat color in swamp buffalo. Anim. Genet..

[128] Iannaccone M., Cosenza G., Pauciullo A., Martino G., Capparelli R. (2018). The SNP g.4667G>A at 30-UTR of IFNG gene is associated with susceptibility to bovine tuberculosis in Mediterranean water buffalo (Bubalus bubalis). Anim. Genet..

[129] El-Halawany N., Abd-el-monsif A.S., Al-Tohamy A.F., Hegazy L., Abdel-Shafy H., Abdel-Latif M.A., Ghazi Y.A., Neuhoff C., Salilew-Wondim D., Schellander K. (2017). Complement component 3: Characterization and association with mastitis resistance in Egyptian water buffalo and cattle. J. Genet..

[130] Othman O.E., Khodary M.G., El-Deeb A.H., Hussein H.A. (2018). Five BoLA-DRB3 genotypes detected in Egyptian buffalo infected with Foot and Mouth disease virus serotype O. J. Genet. Eng. Biotechnol..

[131] Abdi Z., Rafat S.A., Moghaddam G., Hajikolaii M. (2015). A Review on the Polymorphism of Genes/Markers Responsible for Genetically Brucellosis Disease Resistance in Water Buffalo.

[132] Ganguly I., Sharma A., Singh R., Deb S.M., Singh D.K., Mitra A. (2008). Association of microsatellite (GT) n polymorphism at 3′ UTR of NRAMP1 with the macrophage function following challenge with Brucella LPS in buffalo (Bubalus bubalis). Vet. Microbiol..

[133] Garry A.L., Schutta C. (2010). Natural resistance against brucellosis: A review. Open Vet. Sci. J..

[134] Di Stasio L., Albera A., Pauciullo A., Cesarani A., Macciotta N.P., Gaspa G. (2020). Genetics of Arthrogryposis and Macroglossia in Piemontese Cattle Breed. Animals.

[135] Li Z., Lu S., Cui K., Shafique L., Rehman S.U., Luo C., Wang Z., Ruan J., Qian Q., Liu Q. (2020). Fatty acid biosynthesis and transcriptional regulation of Stearoyl-CoA Desaturase 1 (SCD1) in buffalo milk. BMC Genet..

[136] Meuwissen T., Hayes B., Goddard M. (2013). Accelerating Improvement of Livestock with Genomic Selection. Annu. Rev. Anim. Biosci..

[137] Moaeen-ud-Din M., Bilal G. (2016). Genomic selection of Nili-Ravi buffalo: A choice for buffalo breeders. Buffalo Bull..

